# Pluripotent stem cell strategies for rebuilding the human brain

**DOI:** 10.3389/fnagi.2022.1017299

**Published:** 2022-11-02

**Authors:** Francesco Limone, Joseph R. Klim, Daniel A. Mordes

**Affiliations:** ^1^Department of Stem Cell and Regenerative Biology, Harvard Stem Cell Institute, Cambridge, MA, United States; ^2^Department of Molecular and Cellular Biology, Harvard Stem Cell Institute, Cambridge, MA, United States; ^3^Stanley Center for Psychiatric Research, Broad Institute of MIT and Harvard, Cambridge, MA, United States; ^4^Leiden University Medical Center, Leiden, Netherlands; ^5^Faze Medicines, Cambridge, MA, United States; ^6^Institute for Neurodegenerative Diseases, Department of Pathology, University of California, San Francisco, San Francisco, CA, United States

**Keywords:** neurodegeneration, aging, cell replacement therapies, regenerative medicine, pluripotent stem cells, developmental neuroscience, brain regeneration, neurological diseases

## Abstract

Neurodegenerative disorders have been extremely challenging to treat with traditional drug-based approaches and curative therapies are lacking. Given continued progress in stem cell technologies, cell replacement strategies have emerged as concrete and potentially viable therapeutic options. In this review, we cover advances in methods used to differentiate human pluripotent stem cells into several highly specialized types of neurons, including cholinergic, dopaminergic, and motor neurons, and the potential clinical applications of stem cell-derived neurons for common neurodegenerative diseases, including Alzheimer’s disease, Parkinson’s disease, Huntington’s disease, ataxia, and amyotrophic lateral sclerosis. Additionally, we summarize cellular differentiation techniques for generating glial cell populations, including oligodendrocytes and microglia, and their conceivable translational roles in supporting neural function. Clinical trials of specific cell replacement therapies in the nervous system are already underway, and several attractive avenues in regenerative medicine warrant further investigation.

## Introduction

Age—it’s the one mountain you can’t overcome, and as the average life expectancy extends into the eighth decade, neurodegenerative diseases are becoming increasingly prevalent. Despite their increasing incidence, preventative or disease-modifying strategies for these emotionally and financially draining disorders are lacking. Due to the fundamental lack of regeneration within the central nervous system (CNS), neurodegenerative diseases relentlessly attacking discrete populations of neurons are excellent candidates for cell replacement therapies. Here, we review the current prospects on the application of pluripotent stem cell-derived cell types for the treatment of neurodegenerative disease.

Pluripotent stem cells provide a uniquely scalable source of functional somatic cells, including cells of the CNS, that can potentially replace damaged or diseased tissues. Although prospects for using stem cell derivatives seemed fanciful at the start of the millennium, approximately two decades later several clinical trials using cellular products of pluripotent stem cells are underway or about to reach the clinic (Gage and Temple, 2013; Kimbrel and Lanza, 2015; Steinbeck and Studer, 2015; Trounson and DeWitt, 2016). This progress has been facilitated through the development of robust methods for converting human pluripotent stem cells into the specific cell types that are lost in disease. Most techniques are based on fundamental principles learned from developmental biology and aim to recapitulate cell fate determination pathways in the culture dish, and these methods have been thoroughly reviewed elsewhere (Tao and Zhang, 2016). More recently, exogenous over-expression of transcription factors (TFs) has provided an alternative route to directed differentiation methodologies for generating specific classes of neurons. When appropriate, we will highlight both approaches that advance the field toward producing defined cellular populations, which are the ideal candidate for cell replacement therapies.

**FIGURE 1:**
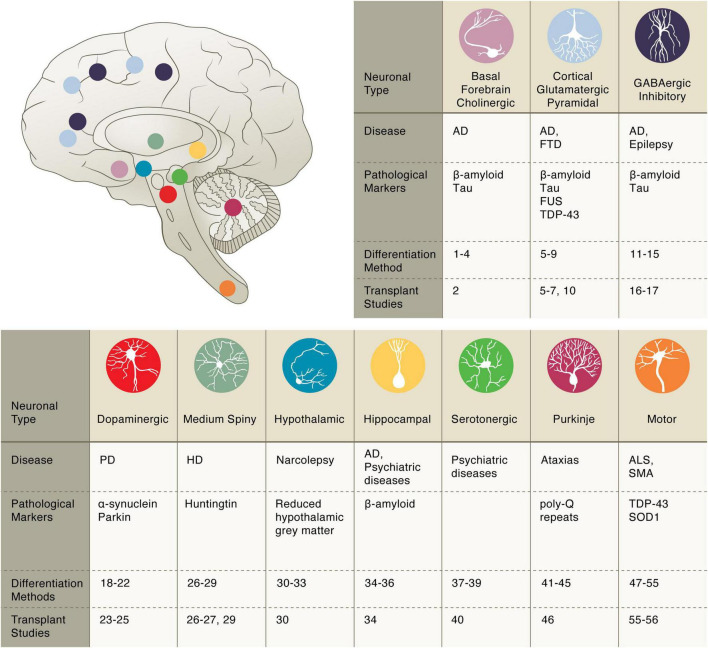
Pluripotent stem cell differentiation strategies for producing vulnerable neuronal cell types. Basal forebrain cholinergic: diff. 1–4 Bissonnette et al., 2011; Liu et al., 2013a,b; Hu et al., 2016; transpl. 2 Liu et al., 2013a. Cortical glutamatergic: diff. 5–9 Espuny-Camacho et al., 2013; Zhang et al., 2013; Cao et al., 2017; Qi et al., 2017; Nehme et al., 2018; transpl. 5–7, 10 Espuny-Camacho et al., 2013; Zhang et al., 2013; Espuny-Camacho et al., 2017; Qi et al., 2017. GABAergic inhibitory: diff. 11–15 Maroof et al., 2013; Nicholas et al., 2013; Chanda et al., 2014; Sun et al., 2016; Yuan et al., 2018; transpl. 16–17 Cunningham et al., 2014; Anderson et al., 2018. Dopaminergic: diff. 18–22 Cai et al., 2009; Caiazzo et al., 2011; Kriks et al., 2011; Pfisterer et al., 2011; Kim et al., 2021; transpl. 23–25 Grealish et al., 2014; Kikuchi et al., 2017; Wakeman et al., 2017. Medium Spiny: diff. 26–29 Aubry et al., 2008; Berkowitz et al., 2016; Ma et al., 2012; Victor et al., 2014; transpl. 26, 27, 29 Aubry et al., 2008; Berkowitz et al., 2016; Victor et al., 2014. Hypothalamic: diff. 30–33 Merkle et al., 2015; Wang et al., 2015b; Kirwan et al., 2017; Rajamani et al., 2018; transpl. 30 Merkle et al., 2015. Hippocampal: diff. 34–36 Yu et al., 2014; Sakaguchi et al., 2015; Hiragi et al., 2017; transpl. 34 Yu et al., 2014. Serotonergic: diff. 37–39 Lu et al., 2016; Vadodaria et al., 2016; Xu et al., 2016; transpl. 40 Carlsson et al., 2009. Purkinje: diff. 41–45 Muguruma et al., 2015; Wang et al., 2015a; Ishida et al., 2016; Watson et al., 2018; Silva et al., 2020; transpl. 46 Higuera et al., 2017. Motor: diff. 47–55 Amoroso et al., 2013; Hester et al., 2011; Son et al., 2011; Du et al., 2015; Lippmann et al., 2015; Maury et al., 2015; Goto et al., 2017; Klim et al., 2019; Limone et al., 2022; transpl. 55–56 Yohn et al., 2008; Corti et al., 2012.

**FIGURE 2:**
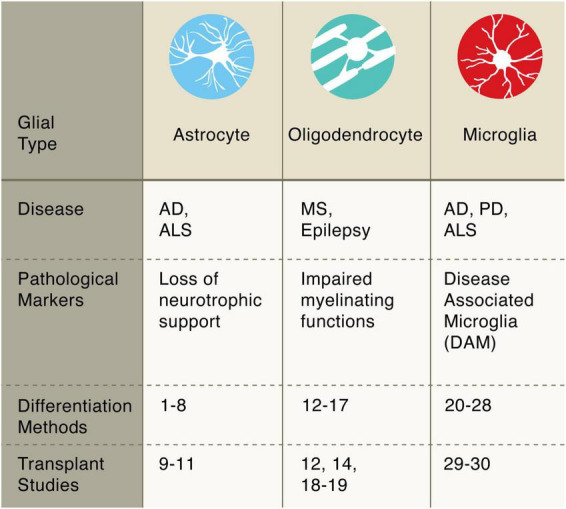
Pluripotent stem cell differentiation strategies for producing glial cells. Astrocytes: diff. 1–8 Krencik and Zhang, 2011; Shaltouki et al., 2013; Tcw et al., 2017; Canals et al., 2018; Li et al., 2018; Tchieu et al., 2019; Barbar et al., 2020; transpl. 9–11 Lepore et al., 2008; Song et al., 2017; Baloh et al., 2022. Oligodendrocytes: diff. 12–17 Wang et al., 2013; Douvaras et al., 2014; Douvaras and Fossati, 2015; Ehrlich et al., 2017; García-León et al., 2018; Marton et al., 2019; transpl. 12, 14, 18–19 Wang et al., 2013; Douvaras et al., 2014; Thiruvalluvan et al., 2016; Windrem et al., 2020. Microglia: diff. 20–28 Muffat et al., 2016; Abud et al., 2017; Douvaras et al., 2017; Haenseler et al., 2017; Pandya et al., 2017; Takata et al., 2017; Chen et al., 2021; Limone et al., 2021; Dolan et al., 2022; transpl. 29–30 Svoboda et al., 2019; Xu et al., 2020.

In this review, we summarize recent progress toward generating specific cell types from human pluripotent stem cells for regenerative medicine. The examples described herein are not intended to be all-inclusive, and readers are encouraged to examine other reviews on the clinical development of stem cell-based therapies (Gage and Temple, 2013; Kimbrel and Lanza, 2015; Steinbeck and Studer, 2015; Trounson and DeWitt, 2016). Rather, we focus on recent biotechnological advances in the derivation of human cells and their application as cell therapies in the field of neurodegeneration (Table 1). These selected studies illustrate the biological concepts, experimental approaches, and therapeutic possibilities of *in vitro* stem cell-derived cells of the neural (Figure 1) and glial (Figure 2) lineages. We conclude our review with a discussion of emerging technologies in the field, current limitations, and remaining challenges for regenerative medicine in translational neurosciences.

**TABLE 1:** Common neurodegenerative diseases characterized by selective vulnerability.

Disease	Prevalence	Main symptoms	Key brain regions affected	Main vulnerable neuronal subtypes	Pathological hallmarks (associated protein)	Therapies (symptomatic treatments)	Regenerative medicine cell-based approaches
Alzheimer’s Disease (AD)	∼5M	Cognitive impairments in memory, language, and behavior	Hippocampus, Basal Forebrain, Locus coeruleus (pons), Cortex	Pyramidal neurons, Cholinergic neurons	Neurofibrillary tangles (tau); neuritic plaques (beta-amyloid & tau)	acetylcholinesterase inhibitors, memantine	Cholinergic neurons, GABAergic Inhibitory neurons
Parkinson’s Disease (PD) and Parkinson’s Disease with Dementia (PDD)	∼1M	Tremor, stiffness, slow movements, autonomic dysfunction, sleep problems, cognitive decline	Substantia nigra (midbrain), locus coeruleus (pons), Cortex (especially the cingulate)	Dopaminergic neurons	Lewy bodies and Lewy neurites (alpha-synuclein)	Levodopa, COMT inhibitors, dopamine agonists, deep brain stimulation	Dopaminergic neurons
Huntington’s Disease (HD)	∼30K	Uncontrolled movements (chorea), neuropsychiatric	Neostriatum, especially caudate (basal ganglia), cortex	Spiny neurons	Intranuclear & cytoplasmic neuronal inclusions (Htt)	Tetrabenazine, neuroleptics (off-label), antidepressants	Spiny neurons
Spinocerebellar Ataxias (SCAs)	∼150K	Difficulty with walking and speech, lack of coordination	Cerebellum, brainstem, spinal cord (dorsal)	Purkinje neurons, pontine nuclei neurons	Intranuclear and cytoplasmic neuronal inclusions (various, e.g., ataxins)	Limited, physical therapy	Purkinje neurons
Amyotrophic Lateral Sclerosis (ALS)	∼20K	Progressive weakness and muscle atrophy	Spinal cord (ventral), brainstem (motor nuclei), and frontal cortex	Upper and lower motor neurons	TDP-43 positive cytoplasmic neuronal inclusions	Riluzole, edaravone	Lower motor neurons

## Parkinson’s disease

Parkinson’s disease (PD) is characterized by the degeneration of several neuronal subtypes, most notably the dopaminergic neurons of the substantia nigra pars compacta (SNpc), located in the ventral midbrain. These neurons project to the dorsal striatum of the basal ganglia and function in motor control, and the loss of these neurons contributes to the movement symptoms observed in the initial stages of PD. Fetal-derived dopamine neurons have had promising clinical benefits for PD patients (Hallett et al., 2014). To avoid the ethical and logistical issues associated with fetal tissue transplants, the application of pluripotent stem cells to generate dopaminergic neurons has been a long-standing goal. Indeed, translational research to bring these specific neurons to the clinic has far exceeded the other cell replacement strategies discussed here and recent advances have extensively been discussed elsewhere (Barker et al., 2017; Kim et al., 2020). In this section we will provide a summary of the most relevant discoveries that led to the first transplantation studies with hiPSC-derived cells that established a road map for the field.

### Dopaminergic neurons

From the initial basic science studies that furnished the directed differentiation strategies of dopaminergic neurons to their large-scale production in GMP-facilities for transplantation studies, the research program for midbrain dopamine neurons has made excellent progress. Several groups developed methods to produce FOXA2/LMX1A-positive midbrain neurons capable of releasing dopamine (Arenas et al., 2015). For example, the Studer group has developed a highly efficient protocol for producing these neurons by combining dual-SMAD inhibition with activation of SHH and FGF8 signaling. The critical step in midbrain specification is the strong activation of WNT signaling achieved using a GSK3β inhibitor (Kriks et al., 2011; Kim et al., 2021). Transcription factors, such as LMX1A, can also be used to enhance directed differentiation approaches (Cai et al., 2009), or for the direct reprogramming of fibroblasts into dopaminergic neurons (Caiazzo et al., 2011; Pfisterer et al., 2011), and combined with cell sorting methods to further enrich for midbrain dopaminergic neurons (Arenas et al., 2015). Preclinical studies demonstrate that human iPS cell-derived dopaminergic neurons are safe and efficacious in both rodent and primate PD models (Kikuchi et al., 2017; Wakeman et al., 2017) with similar efficacy to fetal-derived tissue (Grealish et al., 2014). A number of clinical trials with stem cell-based therapies are currently being planned with their details summarized at a recent consortium meeting (Barker et al., 2017). Although PD patients receiving the stem cell-derived dopaminergic neurons will likely show improvements in movement symptoms, their additional symptoms, including depression, fatigue, visual hallucinations, and sleep disturbances, might persist due to continued degeneration of other neuronal types. This has led to some to propose serotonergic neurons (Lu et al., 2016; Vadodaria et al., 2016; Xu et al., 2016) as an additive cellular therapy for PD (Politis and Loane, 2011). A delicate balance must be struck between dopaminergic and serotonergic neurons, however, as fetal grafts with high levels of serotonergic neurons have been associated with graft-induced dyskinesias in parkinsonian rats (Carlsson et al., 2009).

## Dementia

Neurological conditions involving both memory loss and impaired judgment are classified as dementia (Yue and Jing, 2015). Alzheimer’s disease (AD) is the most common type of dementia in individuals older than 65 years old and the most prevalent neurodegenerative disease (Table 1). The incidence of AD dramatically increases with age, and with the aging US population, it is estimated that approximately 14 million individuals will be affected by 2050. AD often first manifests clinically as impairments with short-term memory, and later affects behavior and language. Current treatments are aimed at ameliorating these symptoms without substantially affecting disease course. Cognitive decline is associated with progressive degeneration of neurons in the limbic system (especially the hippocampus and connected entorhinal cortex), the basal forebrain, and neocortical areas. Histologically, patient brains are characterized by the accumulation of extracellular beta-amyloid depositions and intracellular tau-positive neurofibrillary tangles as well as neuritic plaques that contain both tau within dystrophic neurites and beta-amyloid. Neuropathological studies strongly suggest that AD has well-defined and consistent spatiotemporal pattern of neurofibrillary degeneration, in most cases, that begins in the entorhinal cortex and spreads to pyramidal neurons in the hippocampus and then neocortical areas, with association areas affected sooner and more severely. Currently, there is no effective therapy to block the progression of AD making it a major looming public heath challenge.

### Basal forebrain cholinergic neurons

One of the earliest cell types perturbed by AD is the basal forebrain cholinergic neuron (BFCN). These neurons, which arise from the median ganglionic eminence (MGE) during development, are responsible for various aspects of cognition including learning, memory, and attention. At the molecular level, BFCNs are primary cholinergic neurons and innervate the cerebral cortex, hippocampus, and amygdala, and play critical roles in processing information related to cognitive function (Martinez et al., 2021). Transplantation of fetal cholinergic tissue from rats into the cortex of lesioned primates has been shown to restore memory deficits suggesting a potentially therapeutic roles for these cells (Ridley et al., 1994).

Several methods to differentiate pluripotent stem cells into BFCNs have been described (Bissonnette et al., 2011; Liu et al., 2013a,b; Hu et al., 2016). Typically, first forebrain neural progenitors are obtained and then treated with a SHH agonist and FGF8 to coax the cells into expressing the transcription factors Nkx2.1, consistent with a ventral medial ganglionic eminence (MGE) neural progenitor identity. Subsequent culture of these progenitor cells on glia or treatment with BMP9 then yields a mixture of neurons containing BFCNs (Bissonnette et al., 2011; Liu et al., 2013a,b; Hu et al., 2016). Alternatively, overexpression of the transcription factors Lhx8 and Gbx1 can convert the progenitors into BFCNs (Bissonnette et al., 2011). Cells produced using these methods express markers consistent with a cholinergic identity and exhibit expected electrophysiological profiles. In one study, MGE-progenitor cells transplanted into mouse brains differentiated into neurons, including BFCNs, and formed synaptic connections (Liu et al., 2013a). More importantly, injection of these precursor cells led to learning and memory improvements in lesioned mice (Liu et al., 2013a). Whether these improvements were the specific result of the BFCNs or other cell types remains to be determined but this study provides an important proof-of-principle for the use of stem cell-based therapy to improve cognition.

### Cortical glutamatergic pyramidal neurons

Cerebral cortex development consists of three major processes: cell proliferation, neuronal migration, and cortical organization into multiple well-defined layers. The cerebral cortex contains two major classes of neurons; a majority population of excitatory glutamatergic projections neurons that arise during development from the dorsal telencephalon, which is the developmental precursor to the cerebral cortex, and a minor population of inhibitory interneurons. Through successive waves of neurogenesis, these neurons generate the six layers of the neocortex, which can be further functionally divided based on specific patterns of axonal output and dendritic input. Due to their abundance and ability to project long distances, cortical pyramidal neurons, named for their shape, are able to integrate and send information across the entire nervous system (Bekkers, 2011).

The production of pyramidal neurons from pluripotent stem cells is considered to be a default differentiation fate because it occurs in the absence of exogenous signaling factors (Espuny-Camacho et al., 2013). Inhibiting certain signaling pathways, however, can enhance the yield of cortical glutamatergic neurons by suppressing the emergence of inhibitory interneurons (Cao et al., 2017). More recently, accelerated methods for generating cortical neurons have been reported. One method relies on a cocktail of molecules to both pattern the cells to dorsal forebrain lineage and then inhibit neural stem cell self-renewal to drive neurogenesis, which preliminary data suggests can be timed to achieve the production of neurons of different cortical layers (Qi et al., 2017). Forced expression of the transcription factor Ngn2 in stem cells further accelerates the differentiation to yield very pure populations of glutamatergic neurons (Zhang et al., 2013) that can be enhanced with the addition of developmental cues (Nehme et al., 2018). Transcriptional studies suggest this method favors the production of upper layer neurons, therefore additional methods to achieve the full diversity of cortical layers may still be necessary. After injecting into the postnatal mouse brain, human cortical neurons generated using the methodologies described above displayed proper, long-distance projection patterns and integrated functionally within the host’s circuitry (Espuny-Camacho et al., 2013; Zhang et al., 2013; Qi et al., 2017). Whether they can ameliorate disease phenotypes in animal models remains an unanswered question, but neurons transplanted into a murine AD model display pathological hallmarks of the disease including altered tau biochemistry (Espuny-Camacho et al., 2017).

### GABAergic inhibitory neurons

In both the brain and spinal cord, gamma-aminobutyric acid (GABA)-releasing interneurons are the major class of inhibitory neurons and play crucial roles in modulating neural circuits. There are many distinct subtypes of interneurons that differ in their synaptic connections, expression of neuropeptides, neurotransmitter machinery, and developmental origin with some immature interneurons having the remarkable ability to migrate and disperse long distances to integrate throughout the CNS (Southwell et al., 2014). This integrative property makes interneurons a promising candidate for cell replacement therapies.

Several groups have developed directed differentiation approaches for producing interneurons from human pluripotent stem cells (Liu et al., 2013b; Maroof et al., 2013; Nicholas et al., 2013). These approaches typically inhibit both branches of SMAD signaling as well as WNT signaling using small molecules to achieve robust forebrain induction into cells resembling the MGE, as suggested by expression of the transcription factor Nkx2.1. Careful timing of SHH activation then allows for induction of ventral cell fate in these progenitor cells that develop into GABAergic interneurons as opposed to basal forebrain cholinergic neurons (Liu et al., 2013b). In addition to directed differentiation approaches, transcription factor-mediated inductions of interneurons from stem cells have also been described (Chanda et al., 2014; Sun et al., 2016; Yuan et al., 2018). Minimally, transient expression of ASCL1 and DLX2 can convert stem cells into GABAergic interneurons. When injected into the mouse brain, these cells, migrated, integrated, and matured into a variety of interneuronal subtypes, including expression of the mature subtype markers parvalbumin or somatostatin. Further studies, such as single-cell transcriptomic approaches, are needed to characterize the full repertoire of subtypes of interneurons that can be obtained from pluripotent stem cells. Impressive studies have gone on to show that transplanted interneurons were capable of improving memory (Anderson et al., 2018) and in some cases suppressing seizures and abnormal behaviors in an epileptic mouse model (Cunningham et al., 2014). Based on these promising studies, one biotech company, Neurona Therapeutics, is pioneering the clinical uses for interneuron-based cell therapies for epilepsy and neuropathic pain.

### Hippocampal neurons

Composed of granule and pyramidal neurons, the hippocampus plays a critical role in learning and memory. It is also an area of the brain that deteriorates in AD, additional forms of dementia, and other age-related cognitive declines of distinct etiologies. Interestingly, in addition to the subventricular zone, the dentate gyrus of the hippocampus is a unique site of adult neurogenesis (although the absolute rate of neurogenesis remains controversial). Therefore, incorporation of immature stem cell-derived neurons into existing neural circuity beyond embryonic development is a hopeful prospect.

To generate hippocampal neurons, stem cells are patterned to dorsal forebrain progenitors by inhibiting both branches of the SMAD signaling as well as factors to promote WNT and SHH signaling. Subsequently, WNT3a is applied along with BDNF to drive the neurogenesis of hippocampal granule neurons (Yu et al., 2014; Sakaguchi et al., 2015; Hiragi et al., 2017). Initial findings indicate concurrent WNT and BMP activation can drive the differentiation of the dorsal forebrain progenitors into pyramidal neurons (Sakaguchi et al., 2015). Rodent transplantation studies with hippocampal neural precursors revealed that the human neurons could integrate into the dentate gyrus (Yu et al., 2014), but it remains to be determined if these xenografts can affect disease-related phenotypes in animal models.

## Huntington’s disease

Huntington’s disease (HD) is caused by a CAG trinucleotide repeat expansion within the coding region of the *HTT* gene, resulting in an extended polyglutamine (polyQ) tract within the Huntingtin protein. The progressive loss of neurons and gross atrophy in the neostriatum (caudate nucleus and putamen) disrupts neuronal circuits involving the basal ganglia and leads to gradually worsening motor impairment and, as additional brain regions are affected, significant cognitive and psychiatric symptoms.

### Medium spiny neurons

Medium spiny neurons that reside in the striatum, contribute to the complex circuits that control movement and are particularly vulnerable in HD. During development, these inhibitory neurons arise from the lateral ganglionic eminence (LGE) and are marked by the expression of DARPP32 (dopamine- and cAMP-regulated phosphoprotein Mr∼32 kDa) (Fjodorova et al., 2015). The relatively specific loss of DARPP32+ medium spiny class of neurons in the neostriatum makes HD a strong candidate for cell replacement therapies. Like for PD, fetal transplants have paved the way for stem cell-derived therapies for HD (Freeman et al., 2000).

Numerous groups have validated directed differentiation approaches for producing medium spiny neurons from stem cells (Aubry et al., 2008; Carri et al., 2012; Ma et al., 2012). Like the methods for producing other inhibitory neurons from the neighboring MGE, combinatorial SHH/WNT signaling modulation induces an anterior-ventral fate. Of note, reduced activation of SHH signaling and the addition of Activin A can favor a LGE fate while inhibiting a MGE fate (Fjodorova et al., 2015). A direct conversion method has also recently been described for transforming fibroblasts into medium spiny neurons, specifically, with a combination of 4 transcription factors (CTIP2, DLX1, DLX2, and MYT1L) and two microRNAs (miR-9/9 and miR-124) (Victor et al., 2014). Whether these direct programming methods can be applied to pluripotent stem cells remains to be determined but could be used to improve the yield of medium spiny neurons from stem cells, which are at best ∼50%. When transplanted into a murine striatum, the neurons integrate into the host circuit and project to the proper anatomical targets. In some cases, the transplanted cells neurons can rescue motor deficits in quinolinic acid, an excitotoxin, striatal-lesioned mice, a model of HD (Carri et al., 2012; Victor et al., 2014). In another study, however, the transplanted cells also resulted in cellular overgrowth (Aubry et al., 2008). Based on these studies, refined purification methods to yield more homogenous neuron populations followed by additional animal model studies seem warranted.

## Ataxias

Spinocerebellar ataxias (SCAs) are a clinically and genetically heterogenous group of neurological disorders associated with impairments in motor coordination due to degeneration of the cerebellum and connected neuronal pathways. Many SCAs are caused by CAG nucleotide repeat expansions within certain genes leading to the production of polyglutamine (polyQ)-containing proteins with putative toxic gain-of-function effects. For instance, an autosomal dominantly-inherited, abnormally long (>33 CAG repeats) trinucleotide repeat expansion within *ATXN-2* results in SCA2 that can manifest with ataxia, loss of neurological reflexes, and Parkinsonian symptoms. Ataxias can be associated with other inherited disorders. For examples, an autosomal recessively-inherited GAA trinucleotide repeat expansions in *FXN*, encoding frataxin, cause Friedrich’s ataxia, which is characterized by progressive ataxia, impaired speech, loss of vibratory and proprioceptive sensation due to degeneration of spinal cord neurons and nerve fiber tracts connecting to the cerebellum. There are no effective treatments for these debilitating and often fatal diseases.

### Purkinje cells

Purkinje cells are large inhibitory GABAergic neurons with extensive dendritic arbors that reside within the hindbrain structure of the cerebellum. As the output neurons of the cerebellar cortex, they project to neurons within deep cerebellar nuclei and play an important role in motor coordination. Until recently, the differentiation of human PSCs into Purkinje neurons remained elusive, perhaps due to their late emergence during development. An initial directed differentiation approach for this cell type required several steps and many factors. First, exogenous factors were employed to stimulate endogenous Wnt1 and FGF8 signaling and promote a midbrain/hindbrain identity, and inhibition of SHH signaling was used to pattern cells toward a dorsal identity (Muguruma et al., 2015; Wang et al., 2015a). Then, the maturation process could be accomplished through several methods: plating precursors on mouse cerebellar slice cultures (Watson et al., 2018), within self-organizing, polarized cerebellar structures (Muguruma et al., 2015), or more recently in a defined basal medium optimized for cell culture (Bardy et al., 2015; Silva et al., 2020). Studies indicate that the stem cell-derived Purkinje cells are susceptible to genetic insults, such as the trinucleotide CAG repeat in *CACNA1A* associated with SCA6 (Ishida et al., 2016), that trigger their selective demise, and that they can also engraft into the mouse cerebellum (Wang et al., 2015a). Although more defined and robust methods are needed before cell replacement therapies should be considered clinically, the initial findings have paved the way for producing this neuronal type that is relevant to many neurological disorders.

## Motor neuron diseases

The specific loss of motor neurons underlies several devastating neurological diseases including amyotrophic lateral sclerosis (ALS) and spinal muscular atrophy (SMA). Both diseases involve the progressive loss of motor function, eventually progressing to fatal paralysis. In nearly all (∼97%) of cases of ALS, motor neurons in both the brain and spinal exhibit pathological changes in the cellular localization of the RNA binding protein TDP-43, which include loss of the normal nuclear localization and the formation of cytoplasmic inclusions (Klim et al., 2021).

### Spinal motor neurons

Motor neurons represent a diverse group of neuronal subtypes and provide the pivotal link between mind and the animation of the body. Generally, there are two types of motor neurons; upper motor neurons that reside in the frontal cortex and project to lower motor neurons, found in the ventral brainstem and spinal cord, which in turn form synapses with the musculature. Decades of developmental studies and genetic analyses have illuminated the molecular underpinnings of lower motor neuron specification during embryo development (Dasen and Jessell, 2009) with the morphological gradients well established (Davis-Dusenbery et al., 2014).

Leveraging this knowledge, stem cell scientists developed methods to generate motor neurons from mouse embryonic stem cells by applying retinoic acid (RA) to caudalize the cells toward a spinal cord (the distal or tail end of the neural tube) identity and activating SHH to ventralize them toward a motor, rather than sensory, identity (Wichterle et al., 2002). Several research groups have advanced these earlier findings to reproducibly convert human pluripotent stem cells into vast quantities of motor neurons (Amoroso et al., 2013; Du et al., 2015; Maury et al., 2015; Klim et al., 2019). These approaches typically rely on neural induction through small molecule dual-SMAD signaling inhibition, in some cases activation of WNT signaling, accelerated neurogenesis through inhibition of FGF or NOTCH signaling, all coupled with MN patterning described above (RA and SHH). Son et al. (2011) have used a large cadre of MN-related transcription factors (Isl1, Ascl1, Myt1l, Brn2, Ngn2, Lhx3, and Neurod1) to directly convert fibroblasts into induced motor neurons. Alternatively, simpler protocols were achieved that used a subset these factors to transform human stem cells into motor neurons (Hester et al., 2011; Goto et al., 2017). Recently, we have also shown that transcription factor-based and small molecule approaches could be combined to yield a highly pure population of cervical-like motor neurons from iPSCs with 100% efficiency through the inducible expression of Ngn2 (neurogenin-2) alone coupled with RA and SHH treatments (Limone et al., 2022). Interestingly, carefully varying the timing of retinoid application has been demonstrated to afford more caudal motor neuron fates (Lippmann et al., 2015), but methodologies for producing upper motor neurons, also known as cortical spinal motor neurons (CSMNs), are still lacking. As degeneration of cortical and spinal cord motor regions occur in ALS, a full array of motor neuron subtypes might be needed as a cell replacement herapy.

So far, motor neuron transplant results have been encouraging. For example pioneering transplant studies demonstrate that mES-derived motor neurons injected into tibial nerve of adult mice can form functional NMJs and ameliorate muscle atrophy (Yohn et al., 2008). Another notable study was able to transplant human iPS cell-derived motor neurons into the ventral horns of an SMA mouse model (Corti et al., 2012). The transplanted motor neurons could survive and engraft into the murine spinal cord and could even ameliorate disease phenotypes and extend the life span relative to those receiving a fibroblast transplant (Corti et al., 2012). These exciting initial studies highlight the need for large animal models for testing motor neuron-based cell therapies.

## Glial cells

Although glia are more abundant than neurons, nuances remain in our understanding of how their exact cellular identities are established and how glial developmental pathways can be recapitulated *in vitro* for cell replacement approaches. Three main types of glia exist in the CNS: astrocytes, oligodendrocytes (OLs), and microglia. In brief, astrocytes are responsible for forming and modulating the blood–brain barrier (BBB) and modifying the chemical microenvironment governing synaptic function. Microglia are the resident immune cells of the CNS that function in synaptic pruning during development, immune surveillance, debris clearance and defense from pathogens. Oligodendrocytes are responsible for myelinating axons in the CNS, thereby maintaining strong electrical connectivity of brain circuitry. Glia have been implicated in almost all neurodegenerative diseases, and their dysfunction in this context are more extensively reviewed elsewhere (Zheng et al., 2018). Glial transplantation for the treatment of neurodegenerative diseases has been explored much less than for neurons, though might be advantageous for ameliorating glial dysfunction as well as mitigating the loss of degenerating neurons by engaging in supportive roles. Like neurons, glial cells can be generated by activating development cues or overexpression of cell type-specific transcription factors. We will discuss a selection of strategies to generate glia and their most promising applications to neurodegenerative diseases.

### Astrocytes

Astrocytes are star-shaped glial cells that reside in both the brain and spinal cord to maintain BBB integrity, regulate nutrient flow, and govern neuronal function. They arise relatively early in neuronal development from radial glial progenitor cells usually after these cells have generated neurons. Broadly, differentiation protocols recapitulate developmental cues (Krencik et al., 2011; Shaltouki et al., 2013) by promoting neuronal stem cell (NSC) identity via dual SMAD inhibition and then gliogenesis with morphogens (Krencik and Zhang, 2011). Promoting gliogenesis after NSC differentiation has traditionally been a slow rate-limiting step in the generation of astrocytes, but recent transgenic and chemical strategies have greatly accelerated this process. Expansion of NSCs with Activin A, Heregulin 1β (Neuregulin1), and IGFI (Shaltouki et al., 2013; Tcw et al., 2017), flow cytometry-based enrichment strategies (Barbar et al., 2020) or overexpression of TFs NFIA and SOX9 can dramatically shorten differentiation protocols (Canals et al., 2018; Tchieu et al., 2019). hPSC-derived astrocyte-like cells can be generated in as little as 30 days and show functional properties similar to primary astrocytes in that they uptake glutamate, promote neurite outgrowth, propagate calcium waves, and retain their identity *in vivo* (Krencik and Zhang, 2011; Shaltouki et al., 2013; Li et al., 2018). Many groups have recently developed methods to increase maturity and function of these cells by differentiating them from 3D structures coupled with cell sorting methods (Barbar et al., 2020).

Studies on ALS and PD animal models are laying the foundation for astrocyte transplantation therapies. In ALS models, astrocytes exert toxic gain-of-function effects that can act in a cell non-autonomous manner to contribute to motor neuron degeneration (Di Giorgio et al., 2007, 2008; Meyer et al., 2014; Hall et al., 2017). For instance, mice expressing human mutant SOD1 in astrocytes in addition to neurons had reduced to survival compared to mice only expressing mutant SOD1 in neurons, in other words, a wild-type astrocyte microenvironment may promote motor neuron survival (Bataveljić et al., 2012). Focal transplantation of glial-restricted NPCs (Neuronal Progenitor Cells) into the cervical spinal cord of SOD1 transgenic rats during disease progression extended survival and decreased motor neuron death, in part due to the partial rescue of GLT1 expression in astrocytes (Clement et al., 2003). Clinical trials are ongoing to prove the efficacy of transplanted PSC-derived astrocytes to boost neuronal survival and slow disease progression. For instance, a phase 1/2a trial in a small cohort of ALS patients (NCT02943850) has shown that a single injection of human NPCs engineered to produce glial cell line-derived neurotrophic factor (GDNF) into the spinal cord is safe, and viable grafts differentiated into astrocytes that may be neuroprotective through increased GDNF production (Baloh et al., 2022).

Transplantation studies for PD also showed promising results. Co-transplantation of primary fetal NPCs and rat astrocytes increased long-term engraftment of mature midbrain dopaminergic neurons and increase anti-inflammatory markers in the brains of PD rats (Lepore et al., 2008). Transplantation of primary astrocytes into the SNpc increase synaptosomal dopamine uptake in the striatum, reduce ROS stress, and improved motor deficits of pharmacologically-induced PD rats (Song et al., 2017). These observations suggest hPSC-derived astrocytes may be used to slow disease progression and complement dopaminergic neuron transplantation.

### Oligodendrocytes

Similar to astrocytes, oligodendrocytes are derived in development after neurogenesis. In both the forebrain and the spinal cord, oligodendroglial progenitor cells (OPCs) are generated from Nkx2.1^+^, SHH-derived progenitors and their differentiation is regulated by TFs Olig1 and Olig2. OPCs have an immense ability to migrate and populate the entire brain and spinal cord where most of them further differentiate into committed, myelinating oligodendrocytes (OLs) while a small subset of them are maintained in a progenitor state. Their great migratory abilities, plasticity and pivotal role in neuronal support render these cells ideal for transplantation studies and replacement therapies.

To generate OLs, hPSCs are first converted to a neural stem cell with small molecules or a neural epithelial identity through SHH activation and are then pushed toward an oligodendrocyte progenitors (OPCs) identity by the addition of PDGF-AA. These OPCs can be matured into OLs by various cocktails of small molecules, often containing IGF-1 and T3 (Wang et al., 2013; Douvaras et al., 2014; Douvaras and Fossati, 2015). Several groups have shown that complete maturation of OPCs into highly myelinating oligodendrocytes can be achieved either by injecting these cells *in vivo* (Douvaras et al., 2014) or by differentiating these cells in 3D structures (Marton et al., 2019). Protocols relying on overexpression of several transcription factors, including OLIG2, NKX6.2, and SOX10, were developed to be faster and similarly efficient (Ehrlich et al., 2017; García-León et al., 2018). García-León et al. (2018) found, however, that overexpression of SOX10 alone in NSCs was the most efficient at generating OLs in as little as 20 days, and the generated OLs were capable of myelinating cortical neurons both *in vitro* and *in vivo*.

Stem cell-derived OLs hold promise for both demyelinating diseases and spinal cord injury. Multiple sclerosis (MS) is a chronic, autoimmune disease characterized by the loss of myelin and associated oligodendrocytes, often in a remitting and relapsing clinical course that results in gradual neurological decline. MS-iPSC-derived OPCs can myelinate the corpus callosum of immunocompromised hypomyelinated (shiver) mice (Wang et al., 2013; Douvaras et al., 2014), offering a potential regenerative route for re-myelination for cases of MS that are resistant to immune-suppressant treatment. Strikingly, human iPSC-derived OPCs can myelinate axons in a non-human primate marmoset model (Thiruvalluvan et al., 2016). Long term transplantation studies in both shiver mice and demyelinating cuprizone treatment also showed that these cells can not only migrate to distal regions of the CNS farther than previously believed but can also improve behavior and motor function in murine models (Windrem et al., 2020). These results highlight the feasibility of an iPSC-derived OL transplantation therapy for MS and perhaps for other demyelinating diseases.

### Microglia

Unlike other glial cells, microglia are immune cells not derived from the neuroectoderm but originate from the embryonic yolk sac in early stages of development and then migrate to the neural tube (Ginhoux et al., 2010; Kierdorf et al., 2013). Chemical differentiation strategies generally generate early myeloid progenitors by isolation of delaminating cells from so-called yolk-sac embryoid bodies (Muffat et al., 2016; Haenseler et al., 2017) or by promoting hematopoiesis with hypoxic conditions and defined medias (Abud et al., 2017). Initial studies used co-cultures of these immature myeloid cells with human neurons or murine brain extracts to generate resident brain-like microglia (Takata et al., 2017). These protocols made scalability challenging so others have devised ways to further push immature myeloid progenitors toward microglia-like cells (MGLs) with defined medias containing M-CSF to generate myeloid cells coupled with CNS-enriched TGF-beta and CNS-specific, CSF1-receptor ligand IL34 to promote a brain-like specification of these myeloid progenitors. Generated MGLs show competence to phagocytose (Muffat et al., 2016; Abud et al., 2017; Douvaras et al., 2017; Haenseler et al., 2017; Pandya et al., 2017; Limone et al., 2021; Dolan et al., 2022) respond to IFN-γ and LPS stimulation via secretion of pro-inflammatory cytokines (Muffat et al., 2016; Abud et al., 2017), and migrate to sites of injury (Muffat et al., 2016). When co-cultured with neurons, MGLs have also been observed to secrete anti-inflammatory and pro-remodeling cytokines (Haenseler et al., 2017). Like for other glial cells, transcription factor-based protocols may offer increased efficiency and decreased time for the generation of microglial-like cells. One study has shown that overexpression of transcription factors CEBPA and PU.1 coupled with CNS-patterning molecules described above can generate Microglia-like cells from human iPSC (Chen et al., 2021) with a second one showing improved efficiency by overexpressing PU.1 from primitive hematopoietic progenitors (Sonn et al., 2022). A recent study has defined a set of six transcription factors for the generation of microglia-like cells at a scale sufficient for genetic screening (Drager et al., 2022). Following the progress in the derivation of specific neuronal populations, it is plausible that newer approaches might find that just a few transcriptional factors could be sufficient, when coupled with small molecules, for the generation of this cell type.

Long term engraftment studies have been rendered difficult by the lack of homology between murine and human CSF1, which is pivotal for long term microglial survival. However, initial studies have shown the feasibility of transplantation of hiPSC-derived iMGLs in humanized mouse models (Svoboda et al., 2019; Xu et al., 2020).

## Technological advances

Directed differentiation approaches have evolved considerably since the initial derivation of neurons from human embryonic stem cells (Zhang et al., 2001). Although defined culture conditions that primarily employ small molecules instead of poorly defined co-culture systems are more robust, modern directed differentiation approaches still tend to yield highly heterogeneous cultures containing the cell type of interest along with developmentally related cells. Direct conversion strategies like the ones described above typically yield more homogenous cell populations, but viral integration could disrupt normal gene expression and thus might not be amenable to clinical applications. Alternatively, the use of cell surface antibodies for sorting different neural populations has been pioneered to enrich for more defined cell populations (Yuan et al., 2011), or dyes that are selectively taken up by specific cells could theoretically also be used to mark specific cell types as has been demonstrated for neural precursor cells (Yun et al., 2012). These advances have led to several of these differentiation protocols being used for modeling neurodegeneration in different cell types *in vitro* (Giacomelli et al., 2022), opening the door to their adaptation to transplantation studies in the future. Additionally, several groups have made significant progress in the development of protocols for the generation of 3D structures containing various CNS cell types (known as brain organoids) that can enhance cell type specification and maturation (Del Dosso et al., 2020). Whether this technology can be translated into reproducible, manufacturable products for transplantation studies remains unclear, though it does offer a myriad of intriguing possibilities for the field.

It is unclear whether nascent, immature neurons or elaborate, mature neurons will integrate more successfully into a degenerating brain to provide therapeutic benefit. Either way, the ability to control the functional maturation of stem cell-derived neurons would benefit many applications. For *in vitro* disease modeling studies, we have found that co-culture of human neurons with murine glial cells effectively increased neuronal activity, but co-culture with non-human cells is not an ideal strategy for cell replacement therapies. Instead, Gage and colleagues have developed a defined neuronal medium, BrainPhys, which better mimics the environment present in healthy human brains and enhances both spontaneous electrical and synaptic activity of human neurons (Bardy et al., 2015). Whether increased activity translates into increased survival after transplantation remains an unanswered but fascinating question.

The process of reprogramming adult cells back to the pluripotent state erases many aspects of aging that put vulnerable cells at risk in the first place (Mertens et al., 2018). Although resetting the biological clock makes disease modeling more challenging, it might rid the newly derived cells from the neurodegenerative stimuli of aging when transplanted. Still, there might be aspects of maturation that are critical for neuronal integration or function. Unlike stem cell-derived neurons, for example, neurons directly converted from adult fibroblasts capture the faithful expression of all tau isoforms detected in adult brains at the proper ratios (Capano et al., 2022). Direct conversion of adult cells to replace lost neurons might therefore be an alternative technology to consider and has even been shown to reverse symptoms of PD in a rodent model by converting midbrain astrocytes to dopaminergic neurons (Qian et al., 2020).

## Limitations and challenges

Induced pluripotent stem cell technology marshaled in the possibility of personalized regenerative medicine using therapies based on an individual’s own cells. To this end, investigators in Japan started a clinical trial to treat age-related macular degeneration using autologous transplants, however, the trial was eventually suspended after treating one patient (Mandai et al., 2017). Several hurdles generate significant headwinds for this type of approach including (1) the time and effort needed to generate iPS cells, (2) genomic instability of pluripotent stem cells, and (3) the cost of personalized therapeutics. Most of these hurdles have several potential solutions that we will describe here briefly.

Despite recent advances, the overall time to move from the collection of fibroblasts via skin biopsy in the clinic, the reprogramming of fibroblasts into PSCs with completion of appropriate quality controls, to the differentiation of individualized stem cells into a personal population of a specific cell type, such as mature motor neurons, remains extensive, and hence possibly beyond the therapeutic window for rapidly progressive neurodegenerative diseases like ALS. To meet the demands of future clinical applications, state-of-the-art technologies for the cryopreservation of differentiated cell types are being tested to provide a ready to go off-the-shelf product (Holm et al., 2010; Nishiyama et al., 2016). Indeed, this approach is being pioneered within the PD cell replacement field, which has demonstrated that cryopreserved iPSC-derived neurons can maintain high viability and the molecular properties of a dopaminergic neuron. Moreover, these cryopreserved cells can be directly transplanted into a rat model of PD to reverse functional deficits (Wakeman et al., 2017).

For cell replacement therapies, even rare proliferating cells are especially worrisome because they could ultimately lead to the growth of tumors. Moreover, genomic instability of pluripotent stem cells has long been a concern for the field as aneuploid cells have readily been observed (Draper et al., 2004). To identify more subtle genetic changes, groups have performed whole-exome sequencing on many of the hES cell lines listed on the US National Institutes of Health registry and reported the acquisition of dominant negative p53 mutations, a mutation associated with many cancers, for several hES cell lines (Merkle et al., 2017), and other genomic changes associated with cancer and tumorigenesis (Merkle et al., 2022). Similar studies have also identified recurrent mutations that can occur during the reprogramming process and subsequent propagation (Pera, 2011). Therefore, thoughtful genetic characterization should be standard before stem cells or any of their derivatives are used in the clinic. This analysis will not only be useful to rule out stem cell lines with potentially dangerous mutations but could also be used after transplant to retrospectively identify the distribution of the donor cells.

To overcome the laborious nature of converting somatic cells into pluripotent stem cells, the New York Stem Cell Foundation has developed an automated platform for the high throughput conversion of skin biopsies into iPS cells (Paull et al., 2015). This high throughput platform can be used in conjunction with synthetic modified RNA to reprogram cells and avoid viral transduction (Warren et al., 2010). Finally, xenofree culture conditions have been developed and are now commercially available for deriving and propagating human pluripotent stem cells (Klim et al., 2010; Chen et al., 2011). Collectively, these innovations will help expedite the large-scale generation of clinical grade iPS cells.

Finally, widely applicable and efficient cell banking methods are needed to meet the demand of cell transplantation therapies. There are ongoing efforts in both Japan and the United States to screen and bank cells for allogeneic transplantations. Estimates from Cellular Dynamics International suggest that top 183 haplotypes could cover 95% of the US population. To gain maximum population coverage and provide social justice (Ellison, 2016), a universal stem cell donor could be part of the banking effort. This tactic proposes to use genetic engineering to reduce immunogenicity by removing the MHC molecules from the surface of the cells while also introducing well-established tolerance-inducing molecules (Riolobos et al., 2013; Han et al., 2019). Ultimately, stem cell banking will facilitate regenerative therapies by providing a common and less costly off-the-shelf cellular materials that can be thoroughly characterized before regular and repeated clinical use.

## Concluding remarks

It’s an incredibly exciting time for stem cell-based regenerative medicine with a number of clinical trials started and more just on the horizon for neurodegenerative diseases, including one for PD (Kimbrel and Lanza, 2015). The International Society for Stem Cell Research (ISSCR) has established an updated set of guidelines (Daley et al., 2016) for the clinical translation of stem cell research to ensure safety and appropriate rigor while avoiding the real and present dangers of unregulated stem cell therapies (Berkowitz et al., 2016).

The demand for neurodegenerative disease therapeutics continues to grow as populations around the globe age. Currently, no pharmacological strategies exist that can significantly alter disease course for neurodegenerative diseases, thus cell replacement therapies remain an attractive avenue of exploration. Although the prospect of using stem cell-derived neurons to treat many of the diseases discussed above remains abstract, the PD clinical trials, grounded on years of fetal transplant studies and animal models with high fidelity, will provide important guideposts as others venture into these uncharted territories. In this review, we highlighted current methodologies for generating therapeutically relevant neuronal and glial cell types. Although directed differentiation strategies for some of these CNS cell types are in their nascent stage, they represent important first steps toward heralding in a new era of cellular therapeutics.

## Author contributions

JK conceived of the review and drafted the figures. DM drafted the table. All authors drafted the original manuscript and read, edited, and approved the submitted version.
